# Study of Visual Quality and Higher Order Aberrations in Early Posterior Capsular Opacification

**DOI:** 10.1155/joph/2414100

**Published:** 2025-07-07

**Authors:** Zhangyi Li, Ji Sun, Bin Xv, Jiayv Zhang, Can Li

**Affiliations:** ^1^Department of Ophthalmology, Chongqing Emergency Medical Center, Chongqing University Center Hospital, The Fourth People's Hospital of Chongqing, Chongqing 400016, China; ^2^Department of Ophthalmology, The First Affiliated Hospital of Chongqing Medical University, Chongqing Key Lab of Ophthalmology, Chongqing Eye Institute, Chongqing 400016, China

**Keywords:** higher order aberrations, intraocular lens, posterior capsular opacification, visual quality

## Abstract

**Purpose:** This study aims to explore the visual quality and characteristics of higher order aberrations (HOAs) in patients with early posterior capsule opacification (PCO), providing a theoretical basis for the clinical assessment of early PCO and the potential benefits of Nd:YAG posterior capsulotomy.

**Methods:** This cross-section observational study included 73 patients (73 eyes) diagnosed as PCO at a tertiary hospital from September 2022 to September 2023. All subjects underwent optometric examinations, OQAS, and iTrace measurements, followed by posterior capsule retroillumination photography after full mydriasis. Images were imported into ImageJ software, selecting a 3-mm diameter central area of the intraocular lens (IOL), and the mean gray value (MGV) of this area was recorded. Statistical analyses were conducted on baseline data, the first PCO follow-up time, logMAR corrected distance visual acuity (CDVA), MGV, objective visual quality parameters, dysfunctional lens index (DLI), and various HOAs parameters.

**Results:** Compared to the control group, the PCO group exhibited statistically significant differences in parameters such as OSI, MTF cutoff, SR, trefoil, Z18, and Z24 (*p* < 0.05); however, logMAR CDVA, HOs total, coma, and spherical aberration did not show significant differences (*p* > 0.05). Compared to the monofocal IOL (MoIOL) group, the multifocal IOL (MfIOL) group had earlier PCO follow-ups, with significantly better MTF cutoff and SR, and significantly lower Z7 and Z10 (*p* < 0.05). Subgroup analysis based on OSI showed significant differences between the two groups in parameters such as logMAR CDVA, MTF cutoff, SR, DLI, HOs total, and coma (*p* < 0.05).

**Conclusions:** Early PCO significantly impacts objective visual quality and HOAs in patients. Early intervention may provide greater visual benefits for patients implanted with MfIOL.

## 1. Introduction

Posterior capsular opacification (PCO) is a major complication following cataract surgery that affects visual acuity and quality [1]. It can lead to symptoms such as decreased vision, monocular diplopia, glare, and even severely affected visual quality which require corrective surgery [2]. Statistically, the incidence of PCO is between 4.7% and 18.6% three years post-surgery and 7.1% and 22.6% after five years [3], with rates in children reaching as high as 100% [4]. Wormstone et al. [5] describe PCO as a complex biological process of injury and repair following cataract surgery, where residual lens epithelial cells (LECs) proliferate excessively due to loss of contact inhibition and migrate to the posterior capsule, undergoing an epithelial–mesenchymal transition (EMT) accompanied by the deposition of the extracellular matrix (ECM), thereby forming PCO [6–8].

Neodymium yttrium–aluminum–garnet (Nd:YAG) posterior capsulotomy, first introduced by Aron-Rosa et al. [9] in 1980, is a straightforward, safe, and effective procedure now recognized as the primary treatment for PCO [10]. However, complications such as secondary glaucoma, macular edema, retinal detachment, vitreous prolapse, intraocular lens (IOL) damage, and anterior chamber hemorrhage have been reported in the literature [11]. Clinically, there is no consensus as to the exact time for performing laser capsulotomy, which is generally assessed based on visual acuity and the degree of PCO opacity [12]. Moderate-to-severe PCO, characterized by dense, widespread opacification of the posterior capsule, significantly impairs vision, causing symptoms like diplopia and glare, where Nd:YAG laser capsulotomy typically results in substantial improvement in visual function [13]. However, cases for early PCO can be different, where only mild opacification of the posterior capsule can be found and VA has not been declined [14]. Further studies have shown that capsulotomy can still benefit these patients [15]. Thus, relying solely on visual acuity and capsule cloudiness for surgical decision-making may not be sufficient.

The objective scatter index (OSI) is the ratio of peripheral light intensity to the central peak intensity, reflecting the transparency of the whole refractive medium and the smoothness of optical interfaces [16]. Research has shown that OSI correlates more strongly with medium opacification than visual acuity and contrast sensitivity [17]. Lu et al. [18] proposed using OSI as an objective indication for laser capsulotomy, finding postoperative improvements in both visual acuity and quality. However, their study included only monofocal IOL (MoIOL) patients, lacking analysis on multifocal IOL (MfIOL) cases. Higher order aberrations (HOAs) significantly impact the imaging of the human optical system, potentially causing discomforts such as glare, halo, and reduced vision under low light conditions [19]. The iTrace Visual Function Analyzer (iTrace) based on the principle of ray tracing is an objective incident-type retinal imaging aberrometer that measures whole-eye, corneal, and intraocular wavefront aberrations [20]. Cinar et al. [19] found that total aberrations and HOAs significantly decreased postoperatively in PCO patients (including AcrySof IQ PanOptix and AcrySof IQ SN60WF groups), although there were no significant differences between the groups before and after surgery. However, their study included only moderate-to-severe PCO patients and did not involve early PCO cases, nor did it explain why a higher proportion of early laser surgeries occurred following presbyopia-correcting IOL implantation.

This study included patients with early postoperative PCO and non-PCO, utilizing objective visual quality analysis equipment such as iTrace and optical quality analysis system (OQAS) II, to explore visual quality and HOAs characteristics in early PCO patients. Furthermore, it compared the differences in aberrations and visual quality between MoIOL and MfIOL in early PCO, analyzing the clinical significance of OSI as an objective indicator for laser capsulotomy, with the aim to provide a theoretical basis for the clinical assessment of early PCO characteristics and potential benefits of laser surgery.

### 1.1. Materials and Methods

This cross-section observational study adhered to the Declaration of Helsinki and was approved by the Ethics Committee of a tertiary hospital. Written informed consent was obtained from each participant.

Seventy-three patients (73 eyes) diagnosed as PCO were enrolled in this study, collected from the ophthalmology department of the First Affiliated Hospital of Chongqing Medical University between September 2022 and September 2023. All patients underwent uneventful cataract extraction combined with nonaspheric IOL (ZCB00 (Johnson & Johnson Vision, Santa Ana, CA, USA) or ZXR00 (Johnson & Johnson Surgical Vision, Inc., Santa Ana, CA, USA)) implantation for more than three months, with logMAR corrected distance visual acuity (CDVA) ≤ 0.3. Exclusion criteria included tear film issues, intraoperative complications, severe PCO, any vitreoretinal or corneal diseases, a postdilation pupil diameter less than 6 mm, and patients with a history of ocular surgery, uveitis, trauma, or systemic diseases affecting vision. Ultimately, 45 eyes with PCO were included in the PCO group, and 25 eyes without opacification were included in the control group.

Baseline demographic information was recorded for all study participants, including name, gender, age, eye (left or right), diagnosis, general medical history, and first PCO follow-up time. A comprehensive eye examination was conducted using a slit lamp to assess the anterior segment structures, while the posterior segment structures were evaluated using an indirect ophthalmoscope. Intraocular pressure was measured using a noncontact tonometer. CDVA was recorded using the Snellen chart and converted to logMAR for statistical analysis.

In a dark room, all examinations were performed by the same operator using the OQAS II (VISIOMETERICS, Spain), strictly adhering to standard operating procedures. To ensure consistency of results, the device was preset with an artificial pupil diameter of 4 mm, and refractive errors ranging from −8.0D to +6.0D in sphere and −0.5D to +0.5D in cylinder were automatically corrected by the device; refractive errors beyond these ranges were manually corrected by the operator using lenses. Prior to examination, patients were instructed to blink, and the operator aimed to complete image acquisition and data measurement in the shortest possible time to minimize measurement errors due to factors such as tear film instability. Each eye was measured three times consecutively, and the average values were recorded. The measured parameters included OSI, modulation transfer function (MTF) cutoff value, Strehl ratio (SR), predicted visual acuity (PVA) 100%, PVA20%, and PVA9%.

Based on double-pass technique, OQAS can directly record images of a point-source object after reflection on the retina and analyze critical optical parameters like OSI, MTF cutoff, SR, PVA100%, PVA20%, and PVA9% [16]. The system is extensively used to assess the transparency of the whole refractive medium, imaging quality of the refractive system, and the continuity of optical interfaces. OSI represents the ratio of peripheral to central peak light intensity on the retina, reflecting the transparency of the whole refractive medium and the smoothness of various interfaces. MTF cutoff indicates the resolution limit of the human eye's MTF curve at a spatial frequency of 0.01, with higher values indicating better visual quality. SR compares the intensity of light in an actual optical system to that of an ideal optical system under the same aperture diameter; it can also be considered as the area under the MTF curve. PVA represents the simulated optical vision at three different contrast levels, calculated based on the double-pass OQAS, reflecting the objective visual acuity of a pure optical system [18].

Examinations were conducted in a dark room by the same skilled technician after dilation (pupil diameter > 6 mm) using the iTrace (Tracey Technologies, USA), adhering to standard operating procedures. Before each examination, patients were instructed to blink, and the operator aimed to complete image acquisition and measurement as quickly as possible. All examinations were measured three times per eye consecutively, and the average values were used. Standard-compliant images were selected for data analysis; these images needed to show all reflected Placido rings clearly and completely, without any missing segments, and with fewer than 10 rejected points. A 3-mm-diameter pupil was selected for aberration analysis, obtaining total HOAs, trefoil, spherical, coma, and aberrations from Z6 to Z27, along with dysfunctional lens index (DLI). DLI is an objective lens performance index based on intrinsic HOAs, pupil size, and contrast sensitivity data, used to evaluate lens opacity and surgical assessments [21].

**Figure 1:**
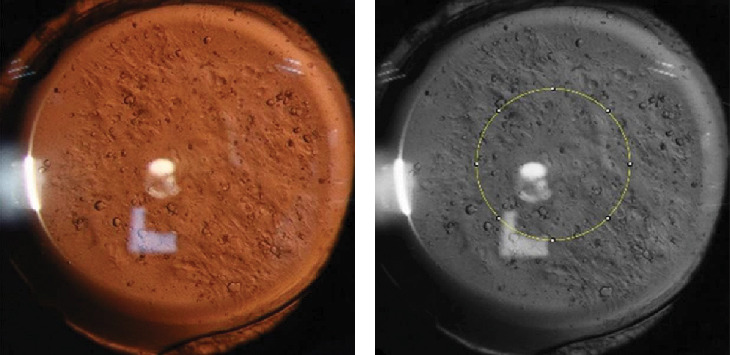
(a) Retroillumination image of PCO. (b) Central 3-mm selection area in ImageJ.

After full dilation, digital retroillumination images of PCO were captured in a dark room using a slit lamp imaging system by the same skilled technician. Patients were positioned comfortably with their chin on the chin rest and forehead against the forehead rest, looking straight ahead with natural eyes open. The operator adjusted the magnification to 16x and set the light band to a slit, focusing coaxially on the posterior capsule using the retro-illumination method to capture images of PCO. After multiple captures, the clearest image was selected and imported into ImageJ 1.8.0_112 to calculate the mean gray value (MGV) in the central 3-mm area, representing the severity of PCO (Figure 1). MGV is derived from image pixel values, with higher values reflecting superior transparency and minimal PCO, and lower values indicating reduced transparency and more severe PCO.

### 1.2. Statistical Analysis

Based on an alpha level of 0.05 and a power of 80%, in a 2:1 ratio between two groups, the required sample size was calculated to be 40 eyes for the PCO group and 20 eyes for the non–PCO group. Statistical analysis was conducted using IBM SPSS Statistics 26.0. Categorical data were expressed as percentages and compared using the Chi-square test; continuous data were tested for normality using the Kolmogorov–Smirnov test, with normally distributed data presented as mean ± standard deviation and skewed data described by median (M) and interquartile range (IQR). Comparisons between groups were made using the Wilcoxon test. Correlations between MGV, OSI, logMAR CDVA, and HOs total were assessed using Spearman's correlation analysis. A *p* value of < 0.05 was considered statistically significant.

## 2. Results

This study initially included 73 patients (73 eyes); however, due to insufficient dilation for retroillumination photography in 2 patients and instability during iTrace assessments in 1 patient (2 in the PCO group and 1 in the non–PCO group), a total of 70 patients (70 eyes) were ultimately included in the analysis. The PCO group comprised 45 patients (45 eyes), including 21 (46.7%) from males and 24 (53.3%) from females, with an average age of 67.63 ± 12.63 year (y); the control group consisted of 25 patients (25 eyes), including 13(52.0%) from males and 12 (48.0%) from females, with an average age of 68.84 ± 8.27 y. No significant differences were observed in gender or age between the two groups (*p*=0.669 and *p*=0.07, respectively).

**Figure 2:**
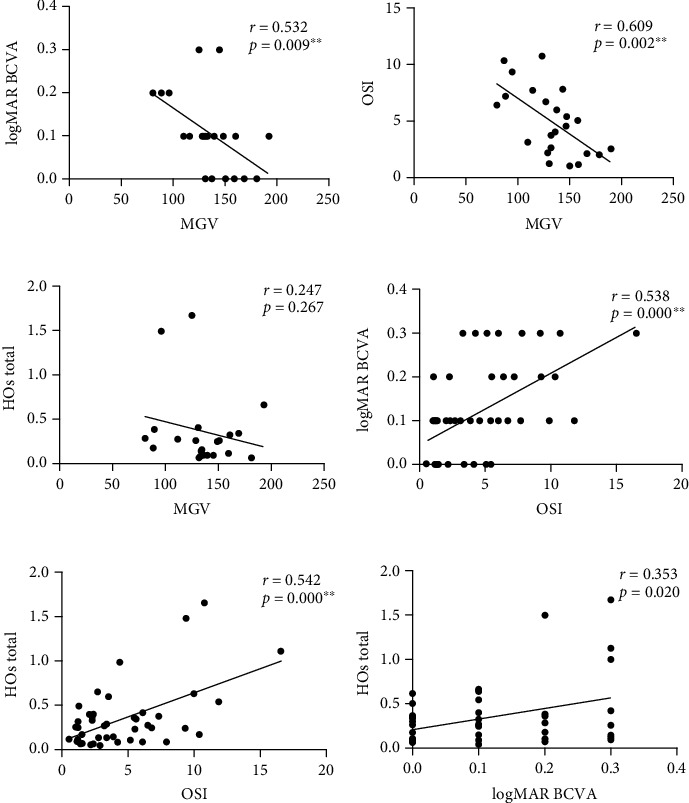
The correlations of visual function parameters in PCO group. Correlation of MGV with logMAR CDVA (a), OSI (b), and HOs total (c). Correlation of OSI with logMAR CDVA (d) and HOs total (e). Correlation of logMAR CDVA and HOs total (f). r, Spearmann's rank correlation coefficient; ^∗∗^*p* < 0.01.

Comparisons between the PCO and control groups revealed statistically significant differences in follow-up time after PCO (*p*=0.002), OSI (*p*=0.001), MTF cut-off (*p*=0.007), PVA 100% (*p*=0.012), PVA 20% (*p*=0.003), PVA 9% (*p*=0.001), and SR (*p*=0.004). Specifically, the follow-up times were significantly later in the PCO group, and the control group exhibited significantly better results in objective visual quality parameters. However, no significant differences were found between the two groups in logMAR CDVA (*p*=0.307) and DLI (*p*=0.864). In the comparison of intraocular HOA, although no significant differences were shown between HOs total (*p*=0.106), Coma (*p*=0.157), and Spherical (*p*=0.704), significant differences were observed in trefoil (*p*=0.017), Z18 (*p*=0.026), and Z24 (*p*=0.040). Other HOAs did not show significant differences (Table 1, Table A1). Further correlation analysis within the PCO group showed significant correlations between MGV and logMAR CDVA (*r* = 0.532, *p*=0.009) and OSI (*r* = 0.609, *p*=0.002). There was also a significant correlation between OSI and logMAR CDVA (*r* = 0.538, *p* < 0.001) and HOs total (*r* = 0.542, *p* < 0.001). Additionally, there was a statistically significant correlation between CDVA and HOs total (*r* = 0.353, *p*=0.020). Notably, no significant correlation was found between MGV and HOs total (*r* = 0.247, *p*=0.267) (Figure 2).

Based on the type of IOL implanted, the PCO group was further divided into MoIOL group (ZCB00) and MfIOL group (ZXR00), comprising 25 and 20 eyes, respectively. The comparison results showed significant differences between the two subgroups in follow-up times after PCO (*p*=0.008), MTF cutoff (*p*=0.005), PVA 100% (*p*=0.009), PVA 20% (*p*=0.011), PVA 9% (*p*=0.007), and SR (*p*=0.015). The MfIOL group had significantly earlier follow-up times and superior results in objective visual quality compared to the MoIOL group. In contrast, there were no significant differences in logMAR CDVA (*p*=0.848), OSI (*p*=0.091), or DLI (*p*=0.102) between the subgroups. Additionally, in the comparison of intraocular HOAs, although there were no significant differences between the MoIOL and MfIOL groups in HOs total (*p*=0.337), coma (*p*=0.493), spherical (*p*=0.123), and trefoil (*p*=0.451), significant differences were observed in Z7 (*p*=0.003) and Z10 (*p*=0.015) (Table 2, Table A2).

Based on OSI values, the PCO group was further subdivided into Group A (OSI ≥ 3.0) and Group B (OSI < 3.0). Analysis results indicated no significant differences in follow-up times after PCO between the two subgroups (*p*=0.4166). However, significant differences were observed in logMAR CDVA (*p*=0.006), MTF cutoff (*p* < 0.001), PVA 100% (*p* < 0.001), PVA 20% (*p* < 0.001), PVA 9% (*p* < 0.001), SR (*p* < 0.001), and DLI (*p*=0.008), with Group B showing superior objective visual quality compared to Group A. In the comparison of intraocular HOAs, significant differences were observed between Groups A and B in HOs total (*p*=0.024), Coma (*p*=0.008), and Z12 (*p*=0.040), Z20 (*p*=0.002), Z26 (*p*=0.031), while no significant differences were found in spherical (*p*=0.487), trefoil (*p*=0.130), or other HOAs (Table 2, Table A3).

## 3. Discussion

PCO is one of the most common complications following cataract surgery, with the occurrence associated with multiple factors, including patient gender, age, surgical techniques, and the material and shape of the IOL [14]. Based on morphological characteristics, PCO can be divided into regenerative and fibrotic types, with each having a distinct impact on visual function [22]. Fibrotic PCO typically originates from the peripheral part of the IOL, whereas regenerative PCO arises from LECs migrating from the equatorial region and spreading extensively across the posterior capsule [23, 24]. These enlarged cystic cells act as small refractors, leading to more severe visual impairment [24]. Moderate-to-severe PCO can significantly reduce visual acuity, while early PCO primarily causes visual quality issues such as decreased contrast sensitivity, glare, chromatic aberration, and stereopsis impairment [25]. However, the effects of early PCO on the optical imaging system of the eye are not yet fully understood.

Visual quality analysis systems provide a deeper understanding of the human refractive system. In this study, early PCO and non-PCO patients were included, and visual quality and HOAs were analyzed using systems such as OQAS II and iTrace. We also explored the differential impact of PCO on the visual quality of MoIOL and MfIOL and assessed the feasibility of using OSI as an objective surgical indication for laser posterior capsulotomy. Due to the limitations in sample size, this study could not perform subgroup analysis based on the morphological categories of PCO. Nonetheless, our research provides objective evidence of visual quality impairment in early PCO patients, particularly in those with MfIOL implants, where mild PCO may lead to earlier visual quality deterioration, highlighting the need for early intervention.

Patients with early PCO generally maintain relatively good visual acuity, but the objective visual quality parameters and HOAs do not perform well. Lu et al. [18] studied the correlation between the degree of PCO opacity, visual acuity, visual function index, and OSI. In a study of 41 patients with preoperative logMAR CDVA < 0.1 undergoing laser posterior capsulotomy, they found no statistically significant change in CDVA before and after surgery (*p* > 0.05), but significant differences were observed in the visual function index score (*p*=0.000) and OSI (*p*=0.000), indicating that early PCO mainly manifests as visual function impairment rather than a significant decrease in visual acuity [15]. In our study, there was no significant difference in CDVA between the PCO and control groups (*p*=0.307), but several objective visual quality indicators such as OSI, MTF cutoff, PVA 100%, PVA 20%, PVA 9%, and SR showed statistically significant differences. Our findings align with previous literature, further validating the characteristics of objective visual quality in early PCO patients.

Aberrations reduce the quality of retinal imaging and limit the eye's visual performance [26]. Aberrations are classified into higher order and lower order, with lower order aberrations having a greater impact on visual acuity but being easier to correct [27]. Lower order aberrations, such as myopia, hyperopia, and regular astigmatism, can be effectively corrected after cataract surgery, whereas HOAs have a more significant impact on visual quality and may lead to optical phenomena such as halos, glare, and blurred vision [28]. Studies have found a correlation between PCO and HOA [19, 29, 30]. In a study of 24 patients with PCO after aspheric IOL implantation, Levy et al. [30] found that the preoperative HOs total was 2.08 μm (SD 2.20), predominantly trefoil, which reached 1.19 μm (SD 1.15). After laser surgery, all RMS values, except for total coma, significantly decreased, including HOs total, tilt, trefoil, quadrafoil, spherical aberration, and total higher order astigmatism. Pupil diameter is an important factor affecting aberrations, with larger pupil diameters associated with greater aberrations [31]. Therefore, this study set the pupil diameter at 3 mm to explore the characteristics of aberrations under the condition of nature pupil. Our study found that trefoil was significantly increased along with certain S5 aberration in the PCO group compared to the control group, consistent with Levy et al. [30], and may be a potential cause of subjective symptoms such as blurred vision and glare in PCO patients.

Different types of IOLs respond differently to early PCO. MfIOLs, designed based on diffraction principles, may be more sensitive to early PCO. Wang et al. [32] reported a higher incidence of laser posterior capsulotomy in patients with MfIOLs after cataract surgery. Our study included PCO patients with aspheric IOL implants, further divided into MoIOL group and MfIOL group. The results showed that while there was no significant difference between the two groups in CDVA and OSI, the MfIOL group exhibited greater sensitivity in objective visual parameters such as the first PCO follow-up time and MTF cutoff, which is possibly due to higher demands for visual quality and lower tolerance for discomfort caused by glare and blurred vision associated with PCO, suggesting that early intervention may be more beneficial for patients with MfIOLs.

Regarding whether MfIOLs increase HOAs after cataract surgery, previous studies have compared the performance of spherical and aspheric IOLs in PCO cases, finding lower HOAs in aspheric IOLs [30]. Additionally, there was no significant difference in HOAs between aspheric and MfIOLs, indicating that aspheric design can reduce postoperative HOA, while diffraction- and refraction-based designs have no significant impact on PCO-induced HOA [33]. To avoid interference from IOLs on aberration measurements, all patients in this study were implanted with aspheric IOLs. Cinar et al. [34] compared the impact of PCO on HOAs after different aspheric IOL implants and found that total aberrations and HOs total significantly improved after laser surgery in both multifocal and MoIOL groups. Our study also found no significant difference in HOA, including HOs total, spherical aberration, coma, and trefoil, between MoIOL and MfIOL groups under small pupil conditions, consistent with previous reports. Additionally, our study found that Z7 and Z10 were significantly increased in the MfIOL group, which may contribute to some optical interference phenomena.

OSI, as an objective method for evaluating PCO, has shown some practical significance in decision-making of laser posterior capsulotomy [16]. Vision has traditionally been used as the main indication for laser capsulotomy, but Zeng et al. [29] found a curvilinear relationship between PCO severity and visual acuity, with some studies suggesting a linear relationship between PCO and intraocular scattering, thus recommending against relying solely on visual acuity to determine the timing of surgery [15]. OQAS compares the total external retinal light to the central retinal light using double-pass technology, obtaining an OSI value to objectively assess the impact of intraocular scattered light [16]. Lu et al. [18] demonstrated a strong correlation between OSI and PCO severity and found that changes in visual function caused by PCO were more sensitive than visual acuity. Our correlation analysis also found significant correlations between OSI and logMAR CDVA, HOs total, and MGV, supporting former researches. Lu et al. [18] have suggested that OSI ≥ 3 can be used as an indication for laser posterior capsulotomy surgery. In this study, PCO patients were compared in subgroups based on OSI values, and the results showed that subjective visual acuity, objective visual quality parameters, and HOAs in group B (OSI < 3) were significantly better than those in group A (OSI ≥ 3). Our study further suggests that an OSI value of ≥ 3 could be a potential surgical indication for laser posterior capsulotomy.

There are also certain shortcomings in this study. The sample size of this study is small, and the subgroup analysis cannot be carried out according to the PCO type. The relevant conclusions still need to be further verified by a larger sample size. This is also the direction of progress of this research. In addition, this experiment did not include cases such as eccentricity and tilt of IOL, and the IOL position also has a certain influence on HOA. In this study, only the intraocular aberration under the 3-mm pupil diameter was analyzed, and the analysis of the aberration under the 5-mm pupil diameter may have different results.

## 4. Conclusions

This study demonstrates that early PCO leads to decreased objective visual quality and increased HOAs in patients. Patients with MfIOL implants may be more sensitive to early PCO, showing a trend toward earlier medical intervention, even under mild conditions, with similar HOAs and better objective visual quality. The clinical assessment of laser posterior capsulotomy based on OSI has practical significance.

## Figures and Tables

**Table 1:** Comparison of visual quality parameters and HOAs (3.0 mm pupil) between the two groups.

Parameters	PCO	Control	*p* value
PCO follow-up time (m)	2.4 (2.0, 3.4)	1.8 (0.6, 2.0)	0.002
LogMAR CDVA	0.1 (0.0, 0.2)	0.1 (0.0, 0.1)	0.307
OSI	3.8 (2.1, 6.4)	1.8 (1.0, 2.7)	0.001
MTF cutoff	15.091 (9.298, 25.391)	25.783 (19.051, 31.918)	0.007
PVA 100%	0.5 (0.3, 0.8)	0.9 (0.6, 1.1)	0.012
PVA 20%	0.3 (0.2, 0.6)	0.6 (0.4, 0.7)	0.003
PVA 9%	0.2 (0.1, 0.3)	0.3 (0.3, 0.5)	0.001
SR	0.088 (0.072, 0.143)	0.136 (0.112, 0.184)	0.004
DLI	5.67 (3.23, 9.47)	7.17 (3.68, 8.50)	0.864
RMS HOs total (μm)	0.263 (0.122, 0.425)	0.343 (0.210, 0.677)	0.106
RMS coma (μm)	0.103 (0.038, 0.206)	0.110 (0.078, 0.294)	0.157
RMS spherical (μm)	−0.015 (−0.036, 0.006)	−0.007 (−0.033, 0.027)	0.704
RMS trefoil (μm)	0.188 (0.142, 0.360)	0.117 (0.069, 0.251)	0.017

*Note:* m, month for follow-up. Values were tested with Wilcoxon signed-rank test.

**Table 2:** Comparison of visual quality parameters and HOAs (3.0-mm pupil) between the subgroups.

Parameters	MoIOL	MfIOL	*p* value	A	B	*p* value
*n*	25	20		27	18	
PCO follow-up time (m)	3.2 (2.3, 4.0)	1.9 (1.3, 2.4)	0.008	2.4 (2.0, 3.4)	2.0 (1.3, 4.7)	0.417
LogMAR CDVA	0.1 (0.0, 0.2)	0.1 (0.0, 0.2)	0.848	0.1 (0.0, 0.2)	0.1 (0.1, 0.2)	0.006
OSI	5.4 (3.1, 7.7)	2.7 (1.9, 4.5)	0.091	—	—	—
MTF cutoff	10.974 (6.283, 17.978)	21.479 (15.014, 32.594)	0.005	9.635 (5.233, 12.811)	28.005 (24.710, 34.753)	0.000^∗∗^
PVA 100%	0.4 (0.2, 0.6)	0.7 (0.5, 1.1)	0.009	0.3 (0.2, 0.4)	1.0 (0.8, 1.2)	0.000^∗∗^
PVA 20%	0.2 (0.2, 0.4)	0.5 (0.3, 0.7)	0.011	0.2 (0.1, 0.3)	0.7 (0.5, 0.8)	0.000^∗∗^
PVA 9%	0.1 (0.1, 0.2)	0.3 (0.2, 0.4)	0.007	0.1 (0.1, 0.2)	0.4 (0.3, 0.5)	0.000^∗∗^
SR	0.076 (0.062, 0.110)	0.114 (0.086, 0.178)	0.015	4.590 (2.205, 8.825)	8.585 (5.333, 9.953)	0.000^∗∗^
DLI	5.22 (3.10, 8.81)	7.79 (4.25, 10.00)	0.102	5.67 (3.23, 9.47)	7.17 (3.68, 8.50)	0.008
RMS HOs total (μm)	0.263 (0.152, 0.425)	0.268 (0.103, 0.426)	0.337	0.294 (0.165, 0.623)	0.156 (0.087, 0.335)	0.024
RMS coma (μm)	0.107 (0.050, 0.219)	0.102 (0.030, 0.165)	0.493	0.152 (0.079, 0.317)	0.041 (0.025, 0.118)	0.008
RMS spherical (μm)	−0.010 (−0.024, 0.025)	−0.023 (−0.040, −0.007)	0.123	−0.014 (−0.039, 0.023)	−0.020 (−0.029, −0.007)	0.487
RMS trefoil (μm)	0.111 (0.076, 0.289)	0.139 (0.048, 0.237)	0.451	0.148 (0.078, 0.323)	0.099 (0.051, 0.212)	0.130

*Note: n*, number of cases. m, month for follow-up. Group A, OSI ≥ 3.0. Group B, OSI < 3.0. Values were tested with Wilcoxon signed-rank test.

^∗∗^ *p* value < 0.001.

**Table 3:** Comparison of HOAs (3.0 mm pupil) between the two groups.

Parameters	PCO	Control	*p* value
HOs total	0.263 (0.122, 0.425)	0.343 (0.210, 0.677)	0.106
Coma	0.103 (0.038, 0.206)	0.110 (0.078, 0.294)	0.157
Spherical	−0.015 (−0.036, 0.006)	−0.007 (−0.033, 0.027)	0.704
Trefoil	0.188 (0.142, 0.360)	0.117 (0.069, 0.251)	0.017
Z6	0.003 (−0.042, 0.045)	−0.015 (−0.065, 0.041)	0.672
Z7	0.000 (−0.035, 0.040)	0.008 (−0.030, 0.023)	0.883
Z8	0.012 (−0.011, 0.041)	0.000 (−0.026, 0.033)	0.473
Z9	0.022 (−0.007, 0.054)	0.056 (0.014, 0.105)	0.188
Z10	−0.001 (−0.018, 0.031)	−0.005 (−0.013, 0.008)	0.184
Z11	−0.007 (−0.026, 0.017)	0.002 (−0.021, 0.009)	0.932
Z12	−0.020 (−0.037, 0.003)	−0.011 (−0.022, 0.014)	0.213
Z13	0.005 (−0.013, 0.019)	0.001 (−0.009, 0.022)	0.840
Z14	0.012 (−0.009, 0.034)	0.000 (−0.013, 0.034)	0.398
Z15	0.002 (−0.012, 0.011)	0.000 (−0.016, 0.004)	0.556
Z16	−0.004 (−0.014, 0.007)	0.003 (−0.004, 0.009)	0.202
Z17	0.005 (−0.003, 0.015)	0.001 (−0.014, 0.012)	0.213
Z18	0.003 (−0.005, 0.016)	−0.001 (−0.010, 0.002)	0.026
Z19	−0.003 (−0.023, 0.009)	−0.001 (−0.008, 0.006)	0.220
Z20	−0.001 (−0.016, 0.005)	0.000 (−0.008, 0.016)	0.492
Z21	0.002 (−0.003, 0.012)	0.001 (−0.002, 0.003)	0.951
Z22	0.003 (−0.003, 0.014)	0.002 (0.000, 0.007)	0.869
Z23	0.001 (−0.004, 0.009)	−0.002 (−0.006, 0.001)	0.144
Z24	0.003 (−0.005, 0.012)	−0.001 (−0.015, 0.001)	0.040
Z25	0.001 (−0.005, 0.006)	0.001 (−0.004, 0.006)	0.699
Z26	−0.002 (−0.013, 0.005)	0.000 (−0.004, 0.003)	0.175
Z27	0.005 (−0.005, 0.011)	0.002 (−0.003, 0.008)	0.699

*Note:* Values were tested with Wilcoxon signed-rank test.

**Table 4:** Comparison of HOAs (3.0 mm pupil) between the two subgroups MoIOL and MfIOL (3.0-mm pupil).

Parameters	MoIOL	MfIOL	*p* value
HOs total	0.263 (0.152, 0.425)	0.268 (0.103, 0.426)	0.337
Coma	0.107 (0.050, 0.219)	0.102 (0.030, 0.165)	0.493
Spherical	−0.010 (−0.024, 0.025)	−0.023 (−0.040, −0.007)	0.123
Trefoil	0.111 (0.076, 0.289)	0.139 (0.048, 0.237)	0.451
Z6	−0.005 (−0.042, 0.029)	0.010 (−0.039, 0.046)	0.640
Z7	0.022 (−0.011, 0.061)	−0.032 (−0.056, 0.016)	0.003
Z8	0.015 (−0.011, 0.049)	0.003 (−0.011, 0.023)	0.560
Z9	0.033 (0.003, 0.076)	0.011 (−0.009, 0.047)	0.185
Z10	−0.007 (−0.028, −0.000)	0.019 (−0.008, 0.058)	0.015
Z11	−0.015 (−0.033, 0.013)	0.005 (−0.009, 0.018)	0.105
Z12	−0.030 (−0.042, −0.001)	−0.016 (−0.030, 0.005)	0.258
Z13	0.005 (−0.015, 0.017)	0.006 (−0.008, 0.021)	0.424
Z14	0.006 (−0.013, 0.032)	0.020 (−0.003, 0.058)	0.392
Z15	−0.001 (−0.014, 0.007)	0.004 (−0.007, 0.013)	0.458
Z16	−0.004 (−0.018, 0.003)	−0.003 (−0.013, 0.0105)	0.522
Z17	0.007 (−0.001, 0.019)	0.001 (−0.009, 0.009)	0.230
Z18	0.004 (−0.007, 0.010)	0.003 (−0.004, 0.018)	0.500
Z19	−0.003 (−0.023, 0.010)	−0.003 (−0.022, 0.005)	0.846
Z20	−0.002 (−0.017, 0.006)	0.001 (−0.004, 0.005)	0.315
Z21	0.004 (−0.007, 0.018)	0.002 (−0.002, 0.006)	0.430
Z22	−0.001 (−0.005, 0.015)	0.006 (0.002, 0.009)	0.100
Z23	0.001 (−0.006, 0.014)	0.002 (−0.003, 0.008)	0.846
Z24	0.004 (−0.001, 0.010)	0.002 (−0.005, 0.013)	0.530
Z25	0.001 (−0.003, 0.010)	−0.003 (−0.010, 0.005)	0.098
Z26	−0.001 (−0.010, 0.005)	−0.002 (−0.015, 0.002)	0.471
Z27	0.005 (−0.005, 0.011)	0.005 (−0.005, 0.010)	0.855

*Note:* Values were tested with Wilcoxon signed-rank test.

**Table 5:** Comparison of HOAs (3.0-mm pupil) between the two subgroups A and B.

Parameters	A	B	*p* value
HOs total	0.294 (0.165, 0.623)	0.156 (0.087, 0.335)	0.024
Coma	0.152 (0.079, 0.317)	0.041 (0.025, 0.118)	0.008
Spherical	−0.014 (−0.039, 0.023)	−0.020 (−0.029, −0.007)	0.487
Trefoil	0.148 (0.078, 0.323)	0.099 (0.051, 0.212)	0.130
Z6	0.043 (0.015, 0.096)	0.044 (0.019, 0.054)	0.148
Z7	0.051 (0.022, 0.091)	0.025 (0.016, 0.042)	0.720
Z8	0.033 (0.014, 0.067)	0.019 (0.011, 0.050)	0.570
Z9	0.034 (0.018, 0.112)	0.030 (0.007, 0.045)	0.643
Z10	0.033 (0.017, 0.063)	0.016 (0.007, 0.044)	0.668
Z11	0.026 (0.015, 0.060)	0.018 (0.008, 0.031)	0.168
Z12	0.033 (0.022, 0.044)	0.020 (0.013, 0.037)	0.040
Z13	0.017 (0.009, 0.054)	0.018 (0.007, 0.026)	0.144
Z14	0.025 (0.007, 0.067)	0.030 (0.014, 0.053)	0.547
Z15	0.017 (0.006, 0.030)	0.007 (0.004, 0.016)	0.281
Z16	0.013 (0.006, 0.025)	0.007 (0.004, 0.013)	0.531
Z17	0.017 (0.008, 0.032)	0.008 (0.002, 0.013)	0.908
Z18	0.011 (0.006, 0.028)	0.005 (0.003, 0.012)	0.763
Z19	0.016 (0.009, 0.038)	0.010 (0.003, 0.016)	0.880
Z20	0.011 (0.003, 0.022)	0.007 (0.004, 0.020)	0.002
Z21	0.012 (0.006, 0.036)	0.003 (0.001, 0.012)	0.144
Z22	0.013 (0.005, 0.026)	0.005 (0.002, 0.009)	0.237
Z23	0.009 (0.004, 0.020)	0.005 (0.002, 0.011)	0.790
Z24	0.008 (0.005, 0.014)	0.005 (0.003, 0.015)	0.313
Z25	0.007 (0.004, 0.025)	0.005 (0.002, 0.012)	0.899
Z26	0.013 (0.004, 0.026)	0.004 (0.002, 0.012)	0.031
Z27	0.011 (0.006, 0.018)	0.007 (0.005, 0.017)	0.509

*Note:* Group A, OSI ≥ 3.0; Group B, OSI < 3.0. Values were tested with Wilcoxon signed-rank test.

## Data Availability

The data that support the findings of this study are available upon request to the corresponding author. The data are not publicly available due to privacy or ethical restrictions.
